# A method to obtain purified free light chain monomers and dimers from urine samples of patients with multiple myeloma

**DOI:** 10.1007/s12026-022-09314-8

**Published:** 2022-09-14

**Authors:** Laura Caponi, Alice Botti, Nadia Romiti, Aldo Paolicchi, Maria Franzini

**Affiliations:** Dept. of Translational Research and New Technology in Medicine, University of Pisa, Pisa, Italy

**Keywords:** Free light chains, Multiple myeloma, Monomers, Dimers

## Abstract

Antibody light chains are synthesized in excess by plasma cells, and this excess can be secreted into biological fluids as dimers or monomers in various proportions. Structural differences between monomers or dimers of free light chains (FLC) can affect their biological functions and possibly their pathogenicity. They also may exhibit differential immune reactivity, perhaps explaining discrepant quantifications when measured by different immunoreagents. Having purified FLC monomers and dimers available can be useful for studying their properties. Here we propose a simple preparatory procedure to purify FLC monomers and dimers from urine samples of patients with plasma cell disorders. Two representative urine samples containing lambda or kappa FLC were loaded into a nonreducing sodium dodecyl sulfate–polyacrylamide gel electrophoresis (SDS-PAGE). The gel strips containing separate monomers and dimers were excised, electroeluted, and the FLC recovered. The FLC were recovered from SDS-PAGE gel in sufficient amounts to be quantified by UV and two automated nephelometric assays immunochemical. The procedure was found to be simple, reproducible, and with a high yield, thus offering the opportunity to compare different assays. Not all urine samples are suitable for this procedure, but this approach allows for the purification of FLC monomers and dimers from many selected urine samples which maintain their oligomeric organization.

## Introduction

The light chains of antibodies are synthesized in excess by plasma cells and secreted in biological fluids. The biological activity of FLC is multiform [1], and direct toxicity has been hypothesized in specific diseases such as light chain (AL) amyloidosis [2, 3]. Structural differences between FLC monomers or dimers can affect their biological functions and their possible pathogenicity. It is known that FLC act on different cell types, interacting with surface receptors even under physiological conditions or even entering cells, thus influencing their differentiation and function [4]. In this regard, the relative signaling capacity of FLC monomers or dimers is poorly understood, as is the extent to which clonal differences and the oligomerization status of FLC from individual patients affect their pathogenetic potential. Furthermore, the factors influencing the propensity of FLC —even when of monoclonal origin—to produce dimers or monomers in various proportions are largely unknown, and the mechanisms that stabilize dimers, particularly those covalently linked by disulfide bonds, have not been fully elucidated. Early observations showed that FLC with a greater potential for oligomeric disposition are more likely to be associated with renal damage [5]. It has been shown that greater dimerization is present in patients with AL amyloidosis and multiple myeloma than in those with monoclonal gammopathy of uncertain significance, and specific patterns of oligomerization have been observed in intrathecal FLC in patients with multiple sclerosis [6, 7].

Since its introduction, the serum κ and λ FLC test has been widely adopted for the management of patients with plasma cell disease and readily included in the guidelines of the International Myeloma Working Group [8–12]. Unfortunately, the two available automated immunochemical FLC assays (N Latex FLC by Siemens Healthcare and Freelite by The Binding Site srl) occasionally provide discrepant results, especially at high values [13–15], therefore in the latest releases of the guidelines, it was necessary to specify the method used to define the thresholds [11]. The reasons why the different automated FLC tests may give discordant results on the same sample are not clear: the heterogeneity of the analyte, the different formulation of the reagent (polyclonal antiserum or mixture of monoclonals), or a different intrinsic reactivity toward a specific monoclonal protein are elements that may contribute. In addition, standard FLC preparations are not available, so the accuracy of FLC testing is still an unsolved issue [16]. Automated immunometric methods currently in use for measuring serum FLC are also intended for use in urine specimens, but there is not sufficient evidence to suggest that the same immunometric methods can be routinely used on urine specimens for diagnostic or prognostic purposes.

In previous studies, we have already documented a differential immune reactivity of lambda FLC monomers and dimers, separated by size exclusion chromatography, when measured by the two Freelite immunoassays and N Latex FLC on the BNII automated platform [17]. FLC monomers and dimers are found in different proportions in each patient’s serum, and this may explain the discrepancies, at least in part. The different reactivity of FLC tests with monomers and dimers was also observed with the FLC contained in the same calibrators used for commercial tests [18]. Purified FLC monomers and dimers are needed to investigate in detail the reasons for the discrepancy between FLC tests and possibly to select epitopes suitable for the reagents, i.e., those not affected by FLC oligomerization.

Previous studies reported procedures for obtaining purified FLC from biological samples. Based on a procedure described by Liang et al. [19], Kaplan et al. [20] devised an improved procedure, which made it possible to study the biochemical characteristics of FLC precursors in deposition diseases. They extracted dimers and monomers from nonreducing SDS-PAGE gel slices, which were eluted overnight. Since the starting material (human plasma) contained a huge amount of proteins other than FLC, several further purification steps (rerun in gel, blot, and elution from membranes) were required to purify the FLC from contaminating proteins.

Another isolation procedure was proposed by Lavatelli et al. [21]. The authors immunoprecipitated FLC from diluted patient serum using Freelite polyclonal antiserum covalently bonded to agarose beads. These FLC were subsequently characterized by proteomics and mass spectrometry, highlighting the dimerization and post-translational modifications. The specificity of the antisera used for affinity purification ensured the purification of the FLC. However, the use of Freelite antiserum as a binding reactive may have introduced a bias in purification, as its differential binding capacity for dimers and monomers was subsequently demonstrated [18]. In other words, immunoprecipitated FLC could be enriched with monomers or dimers and not represent the native production of FLC.

Finally, a more recent study described a large-scale chromatographic purification procedure of FLC from the urine of patients with advanced renal insufficiency [22]. The authors obtained purified FLC using a three-step procedure (salt precipitation, affinity chromatography with specific resins, and gel filtration). Purification of FLC from urine containing high amounts of other proteins was substantial (56- and 100-fold for lambda and kappa, respectively), but the procedure demonstrated a limited yield of purified FLC that ranged from one-quarter to one-fifth of the initial FLC value measured due to the different steps applied to the sample.

Here we propose a simple preparative procedure to purify separated FLC monomers and dimers from two representative urine samples from patients with plasma cell disorders.

## Methods

### Choice of samples and their pretreatment

Samples consist of residual urine specimens left over from the scheduled clinical follow-up urine test, produced from patients with multiple myeloma. To describe the procedure, we report here the data obtained on two representative urine samples, one containing a high concentration of monoclonal lambda FLC (sample A), which had previously been characterized [17], and the other containing a significant amount of kappa FLC (sample B). The samples were stored at – 20 °C in small aliquots until they were used.

The urine samples were processed as a whole sample or after dialysis to remove UV-absorbing solutes in order to estimate protein content by UV absorption [17].

Dialysis was performed for 24 h at 4 °C against Dulbecco’s phosphate buffered saline (PBS) without Ca^2+^ and Mg^2+^ using a dialysis tube (Sigma) with 14,000 Da cut-off, which was pre-boiled for 10 min in buffer containing NaHCO_3_ 2% and 1 mM EDTA.

### Nonreducing SDS-PAGE, Western Blot (WB), and extraction of FLC from gel by electroelution

For the preparative nonreducing SDS-PAGE, 480 µL of dialyzed urine sample A and 500 µL of dialyzed urine sample B were subjected to nonreducing SDS-PAGE onto precast 12% polyacrylamide gels (Bio-Rad), to maintain covalently linked dimers.

For each sample, two gels were run in parallel: one gel was stained with Coomassie Brilliant Blue R-250 (BioRad), while the other was transferred to a nitrocellulose membrane, as previously described for WB [18]. After determining the exact position of the FLC bands and the position of the contaminating protein bands, a preparative gel of the same size was loaded with 500 µL of urine.

At the end of the run, the horizontal narrow strips of the gel expected to contain monomers and dimers of FLC were separately excised and placed in separate dialysis tubes, each containing 1 ml of modified transfer buffer (Tris–glycine pH 7.4 without methanol). The tubes were placed on an electrotransfer stand (Mini-PROTEAN Tetra system with Mini Trans-Blot module, Bio-Rad), and a constant current of 200 mA was applied for 150 min. After electroelution, the buffer containing the eluted FLC was collected. The purity of the eluted protein was then verified in another SDS-PAGE followed by Coomassie staining and WB.

### Quantification of eluted FLC

The concentration of purified FLC monomers or dimers was evaluated by UV absorption at a wavelength of 280 nm using a UV–vis Cary 50 BIO spectrophotometer (Varian, Agilent). The FLC concentration was calculated assuming a molar extinction coefficient at 280 nm for FLC of 1.45 per 1 mg/mL, which is the molar extinction coefficient for human immunoglobulins.

### Nephelometric measurement

The kappa and lambda monomers and dimers were also quantified by the automated immunochemical assays Freelite (The Binding Site Group Ltd, Birmingham, UK) and N Latex FLC (Siemens Healthineer Diagnostics GmbH, Marburg, Germany) with a BN II nephelometer (Siemens Healthineers Diagnostics, GmbH, Germany). In some cases, in the absence of whole antibodies in the sample, the concentration of the light chains was also measured with an anti-lambda (OWHH09) or anti-kappa (OWHG09) Siemens antiserum not specific for FLC.

### Densitometric scan

Densitometric scan of the gels and peak area calculations were performed using Fiji [23].

## Results

### Protein pattern of the two urine samples

**Fig. 1:**
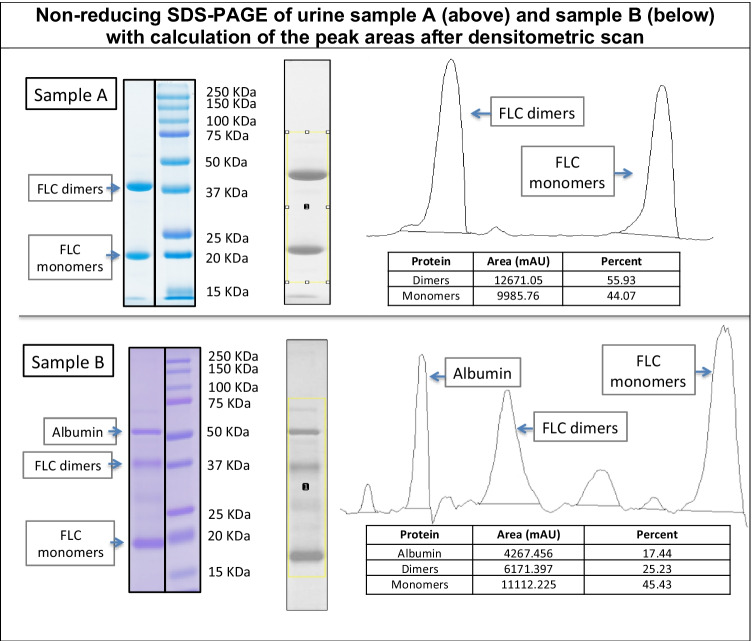
On the left: non-reducing SDS-PAGE of urine samples. The gel was stained with Coomassie. Sample **A** clearly shows monoclonal FLC only. Sample **B** also shows other proteins, with albumin being the most represented. On the right is the corresponding densitometric scan on both samples. The peak areas corresponding to dimers and monomers were calculated for both samples

The urine sample containing lambda FLC (sample A) showed only two protein bands, corresponding to the expected molecular weight for FLC monomers and dimers, while the sample containing kappa FLC (sample B) revealed at least one more intense band of molecular weight (MW) slightly higher than 50 kDa. The Coomassie stained lanes containing lambda or kappa FLC were subjected to densitometric scan in order to verify the relative proportion of dimers and monomers (Fig. 1).

### FLC monomers and dimers: purification, recovery, and quantification

**Fig. 2:**
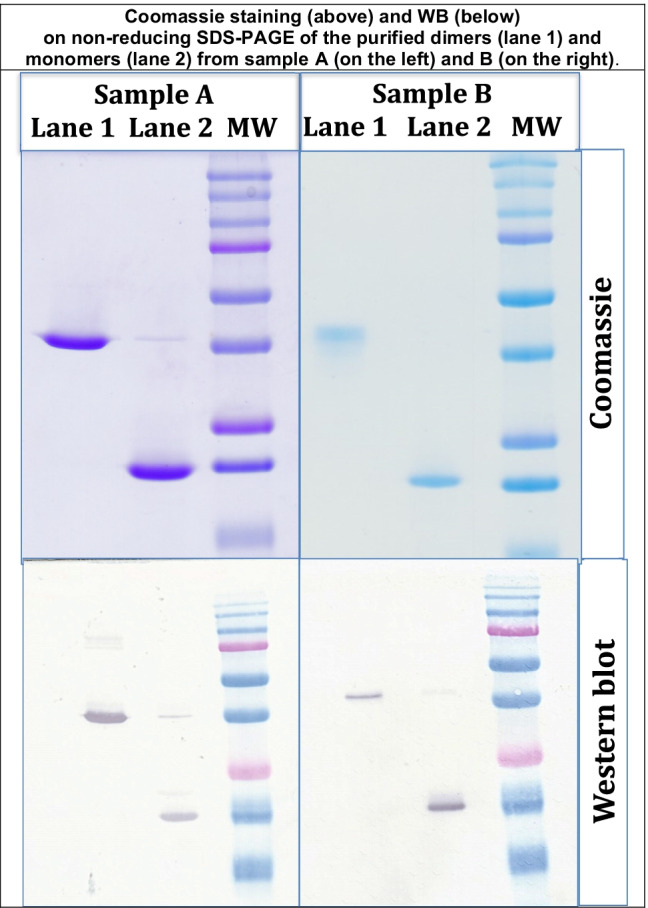
For both samples **A** and **B**, lane 1 shows eluted dimers and lane 2 shows eluted monomers; MW, reference molecular weights (the same as in Fig. 1). Comassie staining is shown above; Western blot is shown below. The apparent MW is around 20 kDa for monomers and between 37 and 50 kDa for dimers

FLC dimers and monomers purified from separating gel by electroelution appeared as homogeneous bands in the gel (Fig. 2).

The electroeluted gel fragments in both cases did not stain significantly with Coomassie, confirming complete elution of the proteins and the high recovery. A subsequent SDS-PAGE of the electroeluted FLC, followed by Coomassie staining and WB, confirmed the purity of the lambda and kappa dimers and monomers.

The recovered FLC were finally quantified. As assessed by UV absorption, for sample A, 500 µg of FLC dimers and 530 µg of FLC monomers were recovered, while for sample B, 87 µg of FLC dimers and 174 µg of FLC monomers were recovered. The proportion of lambda and kappa dimers and monomers recovered roughly corresponds to the intensity of the Coomassie stain in the dialyzed samples. A comparison between the quantification of FLC monomers and dimers by UV absorbance and nephelometric assays was performed. The purified monomers and dimers obtained from the two urine samples were quantified by Freelite and N Latex FLC assays on the BN II nephelometer. In relation to the amount calculated based on UV absorption, for the purified lambda FLC, the Freelite test quantified FLC monomers (672 vs 554 µg/mL) and dimers approximately twice (958 vs 456 µg/mL) in a fairly similar way. The N Latex FLC test measured dimers about one and a half times (745 vs 456 µg/mL), while monomers were largely overestimated (8780 vs 554 µg/ml, about 16 times). The measurement of monomers and dimers purified with the nonspecific anti-lambda antiserum for FLC underestimated the FLC in both cases.

For the kappa sample, compared to the values obtained from the UV absorbance, the Freelite test measured a lower concentration of FLC (seven times lower, 20.8 vs 150 µg/µL), while the monomers were strongly overestimated (674 vs 138 µg/µL, nearly five times). N Latex FLC also largely underestimated dimers (6.3 vs 150 µg/mL), while monomers were measured one and a half times higher (216 vs 138 µg/mL). For both oligomeric forms, the nonspecific anti-kappa antiserum for FLC demonstrated insufficient sensitivity. Table 1 summarizes all the data.

**Table 1:** Recovery of dimers and monomers from the dialyzed urine sample A (above) and sample B (below) and measurement of light chain concentration measured by three nephelometric assays

Recovery and quantification of dimers and monomers from samples A and B					
	Light chain concentration calculated on UV (µg/mL)	Recovery (µL/µg)	Light chain concentration measured by nephelometer (µg/mL)		
			Freelite	N Latex FLC	Non-specific LC
Sample A (containing lambda FLC)					
Purified dimers	456	1096**/**500	958	745	226
Purified monomers	554	956**/**530	672	8780	395
Sample B (containing kappa FLC)					
Purified dimers	150	580**/**87	20.8	6.3	< 71.6
Purified monomers	138	1216**/**174	674	216	< 71.6

Since sample A contained only lambda FLC and no other proteins, the dialysis of the sample enabled the estimation of its FLC content—2400 µg/mL—by UV absorption. Therefore, we were able to estimate the yield of the electroelution: the total amount of FLC in the sample loaded (480 µL of dialyzed urine) corresponds to 1150 µg of lambda FLC, and consequently, the yield of the electroelution (1030 µg of total lambda FLC recovered) corresponds to a recovery of 89.6%. We performed the purification procedure several times on different quantities of the same dialyzed sample A, and therefore, with a different load of FLC to be purified, the recovery of monomers and dimers from the gel varied from 69.9 to 99.5%, with an average of 84.2%. Table 2 details the yield of the runs.

**Table 2:** Yields of the recovery of dimers and monomers from the dialyzed urine sample A (containing lambda FLC) in seven different runs

Yields of different runs of electroelution for sample A						
Run	µl loaded	Total µg loaded	Recovered DIMER µg	Recovered MONOMER µg	Total FLC recovered	Percentage of recovery
1	250	600	322	275	597	99.5
2	300	720	380	283	663	92.1
3	300	720	341	208	549	76.3
4	480	1150	500	530	1030	89.6
5	486	1166	531	491	1022	87.7
6	500	1200	496	343	839	69.9
7	500	1200	590	301	891	74.3

## Discussion

We have described a simple procedure to obtain purified FLC dimers and monomers, useful for studying the various characteristics of FLC, both those that affect their biology and those that may help to explain the discrepant results obtained in sera with different immunochemical techniques, observed in patients with multiple myeloma. The procedure described here takes advantage of the high concentration of FLC in selected urine samples from patients with plasma cell disorders, which do not require the demanding and time-consuming purification steps necessary to extract FLC from plasma samples.

The procedure cannot be applied to all patients, especially when a large amount of other proteins is present in their urine samples, as for example, in the presence of kidney damage, but many urine samples from patients with multiple myeloma contain relatively pure FLC with low amounts of other proteins, mainly albumin and/or whole monoclonal components, which are easily separable from FLC by SDS-PAGE electrophoresis. This procedure, when performed under non-denaturing conditions, allows the separate purification of the FLC monomers and dimers from the urine sample. To increase the number of suitable samples to process, we are working on a possible sample pretreatment to enrich the FLC by removing most of the other proteins.

We can confirm the differential reactivity on well-purified lambda dimers and monomers already observed on monomers and dimers obtained by chromatographic separation [10], extending the evaluation also to the FLC kappa. It is not known whether overestimation or underestimation is a common feature of a specific reactive on all dimers or on all monomers. We have started a study that measures purified dimers and monomers from different urine samples containing monoclonal FLC, and the preliminary results suggest that monomers and dimers from different samples, even though belonging to the same type (kappa or lambda), can react very differently with the immunochemical reagents (unpublished data).

The high purification yield is a key point of this process, which makes it possible to obtain relatively high quantities of FLC that maintain their oligomeric organization without structural alterations deriving from variations in the thiol-disulfide state of the protein.

Moreover, the high level of purification of monomers and dimers reached allows FLC to be obtained in sufficient quantities to separately verify their reactivity with respect to the FLC immunochemical tests. This can help to understand to what extent the occasional discrepancy observed between results obtained with different reagents on the same sample depends on the different quantitative ratio of monomers and dimers and to what extent it depends on the immunoreactivity of the monoclonal protein of each specific patient.
